# Galectin-3 plays a key role in controlling infection by *Toxoplasma gondii* in human trophoblast cells and human villous explants

**DOI:** 10.3389/fcimb.2024.1459810

**Published:** 2024-11-25

**Authors:** Luana Carvalho Luz, Mayara Ribeiro, Samuel Cota Teixeira, Guilherme de Souza, Marina Paschoalino, Daniel Pereira Sousa, Alessandra Monteiro Rosini, Natalia Carine Lima dos Santos, Rafael Martins de Oliveira, Joed Pires de Lima Júnior, Izadora Santos Damasceno, Marcos Paulo Oliveira Almeida, Matheus Carvalho Barbosa, Celene Maria de Oliveira Simões Alves, Claudio Vieira da Silva, Bellisa Freitas Barbosa, Eloisa Amália Vieira Ferro

**Affiliations:** ^1^ Laboratory of Immunophysiology of Reproduction, Institute of Biomedical Sciences, Universidade Federal de Uberlândia, Uberlândia, MG, Brazil; ^2^ Department of Pharmacology, Institute of Biomedical Sciences, Universidade Federal de Uberlândia, Uberlândia, MG, Brazil; ^3^ Trypanosomatid Laboratory, Department of Immunology, Institute of Biomedical Sciences, Universidade Federal de Uberlândia, Uberlândia, MG, Brazil

**Keywords:** maternal-fetal interface, congenital toxoplasmosis, placental, Gal-3, immune response

## Abstract

Galectin-3 (Gal-3) is a β-galactoside-binding lectin expressed in cells of the placental microenvironment. This lectin is involved in various biological processes, such as modulation of the immune system and control of parasitic illness. *Toxoplasma gondii* infection can lead to congenital transmission and cause miscarriages, prematurity and fetal anomalies. However, little is known about the role of Gal-3 in *T. gondii* infection in the placental microenvironment. This study aimed to unravel the underlying mechanisms of Gal-3 during *T. gondii* infection. For this purpose, we promoted the knockdown of Gal-3 expression by using RNA interference (RNAi) in BeWo cells or by using a synthetic inhibitor (GB1107) in human villous explants. We showed that the decreased Gal-3 expression in BeWo cells and human villous explants increases the invasion and proliferation of *T. gondii* probably by downregulating MIF and IL6 levels, highlighting thus the role of this lectin in modulating the immune response. Collectively, our study reveals Gal-3 as a promising target protein during congenital toxoplasmosis.

## 1 Introduction

Galectins are a subgroup of lectins that contain one or two carbohydrate-recognition domains (CRDs) with affinity for glycans containing β-galactoside (Liu and Rabinovich, 2010; Chou et al., 2018). Galectin-3 (Gal-3) is a soluble protein of approximately 30kDa that has a single C-terminal CRD (Challis et al., 2009; Schminkey and Groer, 2014; Brinchmann et al., 2018).

Gal-3 is located in the cytoplasm and nucleus of cells, as well as on the cell surface and in the extracellular matrix (Sciacchitano et al., 2018). Gal-3 participates in several biological processes, such as regulation of growth, proliferation and cell differentiation, apoptosis, signal transduction, and cell-cell interactions (Chou et al., 2018). In addition, Gal-3 participates in immune responses, including acute and chronic inflammatory processes, in the regulation of immune tolerance and also in responses associated with host-pathogen interactions (Maquoi et al., 1997; Blois et al., 2007; Sato et al., 2009; Liu and Rabinovich, 2010).

Gal-3 is expressed in several trophoblastic cell lineages, including villous and extravillous trophoblasts, such as the BeWo and HTR-8/SVneo lineages, respectively, and in human chorionic villi from the first and third trimesters of pregnancy (Maquoi et al., 1997; Hutter et al., 2016; Bojić-Trbojević et al., 2019; Xiao et al., 2019). In the placental microenvironment, Gal-3 plays a crucial role in the embryo’s interaction with the endometrium during implantation, and its expression by endometrial cells is regulated by human chorionic gonadotropin (HCG) (Yang et al., 2011, 2013).

It has been shown that Gal-3 is involved in the interaction between trophoblastic cells and the extracellular matrix during the placentation process (Maquoi et al., 1997). In this context, low expression, or absence, of Gal-3 compromises embryo development by interfering with the interaction of placental villi with the endometrium (Freitag et al., 2020).

Gal-3 is essential for the recruitment of immune effector cells, a vital mechanism for resisting infections, and plays a significant role in managing infections caused by *Trypanosoma cruzi* (Chain et al., 2020; Pineda et al., 2020); and *Leishmania* spp (Oliveira et al., 2021). Gal-3 also plays a significant role during *Toxoplasma gondii* infection (Bernardes et al., 2006; Alves et al., 2010; Debierre-Grockiego et al., 2010; Alves et al., 2013; Liu et al., 2018), an intracellular protozoan parasite, responsible for serious morbidities related to newborns and immunocompromised individuals (Blader et al., 2015; Zhang et al., 2019). Infection during or immediately before pregnancy can result in the vertical transmission of *T. gondii* tachyzoites, which can cross the placenta and invade fetal tissues. Congenital infection can be systemic and particularly severe, leading to spontaneous abortion, stillbirth, fetal death, fetal anomalies, encephalitis, chorioretinitis and infantile disability (Peyron et al., 2017; Aguirre et al., 2019).

Alves et al. (Alves et al., 2010) demonstrated that Gal-3 increases the viability and activation of *T. gondii*-infected neutrophils. Additionally, Gal-3 is essential to produce reactive oxygen species in neutrophils from mice infected with the RH strain of *T. gondii* (Alves et al., 2013). In this context, it has been reported that Gal-3 plays a role in innate immunity by generating a pro-inflammatory profile and by playing a regulatory role in dendritic cells, capable of modulating adaptive immunity (Bernardes et al., 2006). Gal-3 is also associated with parasitism control in mice orally infected with *T. gondii*, by triggering a pro-inflammatory response in the intestine, liver and brain tissues (Bernardes et al., 2006). Furthermore, Gal-3 may play a critical role in regulating cytokine production in *T. gondii*-infected host mice (Liu et al., 2018).

The current literature has revealed the importance of Gal-3 in promoting adequate placental function, as well as its importance during systemic infection by *T. gondii*; however, there are no data in the literature on the functional participation of this galectin during *T. gondii* infection in the placental microenvironment. Herein, we aimed to assess the mechanistic role of Gal-3 during *T. gondii* infection at the maternal-fetal interface.

## 2 Results

### 2.1 Efficient reduction of galectin-3 expression in BeWo cells via shRNA transfection

**Figure 1:**
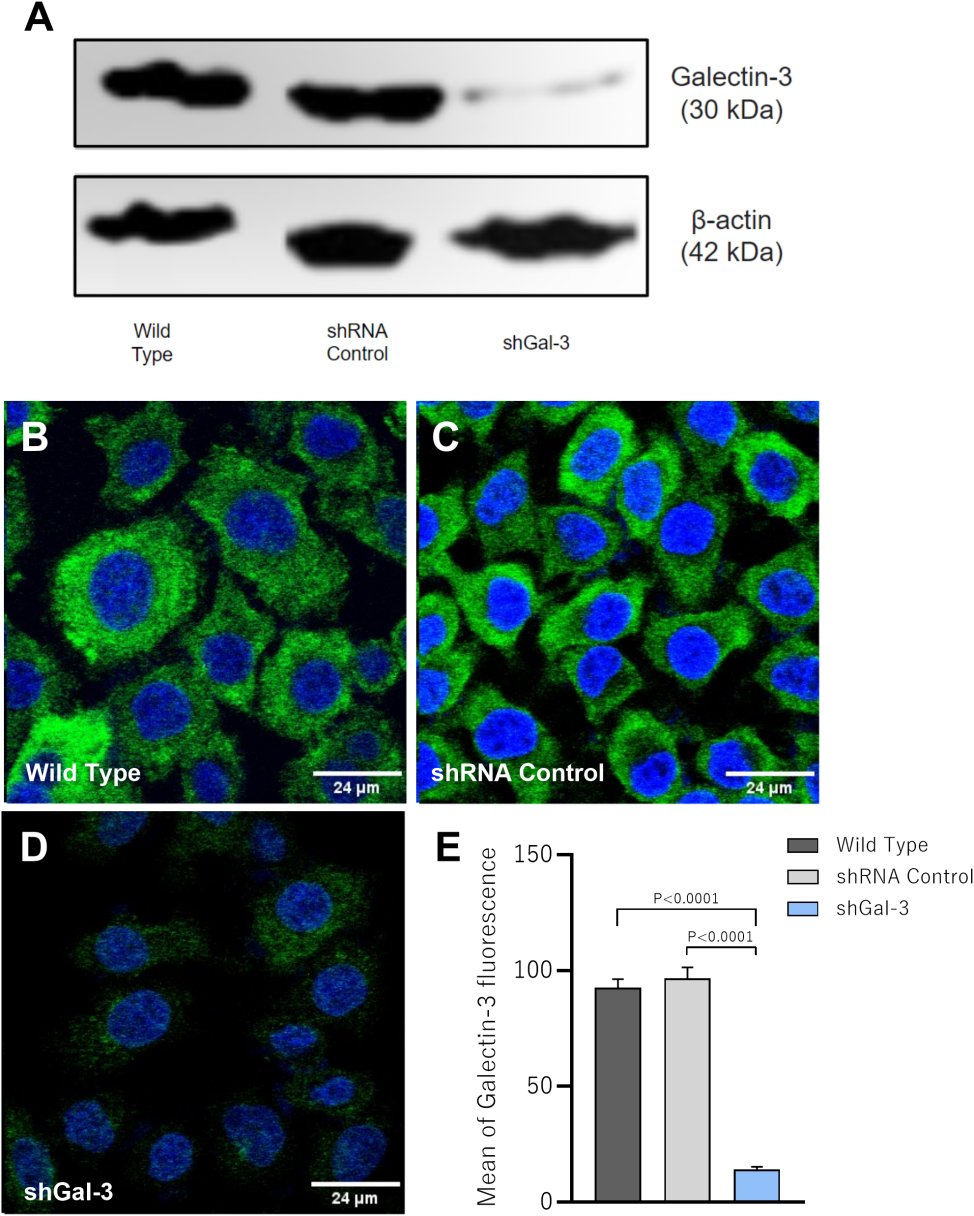
Galectin-3 expression is efficiently reduced in BeWo cells after shRNA transfection. BeWo cells were transfected with Galectin-3 shRNA or control shRNA lentiviral particles. The knockdown of Gal-3 was confirmed by western blotting **(A)** and immunofluorescence assays **(B–D)**. **(E)** Graphical representation of the mean of fluorescence intensity (MFI) of Gal-3 expression. Photomicrographs were captured at 40x magnification. The cell nucleus is labeled with TO-PRO-3 (blue); Gal-3 labeled with Alexa Fluor 488-conjugated is shown green. Scale bar 24µm.

After promoting Gal-3 knockdown in a human villous trophoblast cell line (BeWo cells) using shRNA lentiviral particles, the reduction of Gal-3 expression was confirmed by western blotting and immunofluorescence techniques. We showed that protein expression was partially reduced only in BeWo cells subjected to Galectin-3 shRNA lentiviral particles compared to the control groups (Wild Type and shRNA control); in addition, Gal-3 expression was not altered in BeWo cells submitted to the shRNA control lentiviral particles in comparison with the Wild Type BeWo cells (**Figure 1A**). Regarding the immunofluorescence assay, Wild type (**Figure 1B**) and shRNA control (**Figure 1C**) BeWo cells expressed Gal-3 (green color) homogeneously throughout the cell, while in shGal-3 BeWo cells the detection of the protein was very reduced or almost imperceptible (**Figure 1D**). In this line, the quantification of the fluorescence revealed a significant decrease of the mean of Gal-3 fluorescence in shGal-3 cells compared to both Wild Type and shRNA control (P<0.0001) cells (**Figure 1E**).

### 2.2 Decreased expression of Gal-3 in BeWo cells increased *T. gondii* invasion and intracellular proliferation

**Figure 2:**
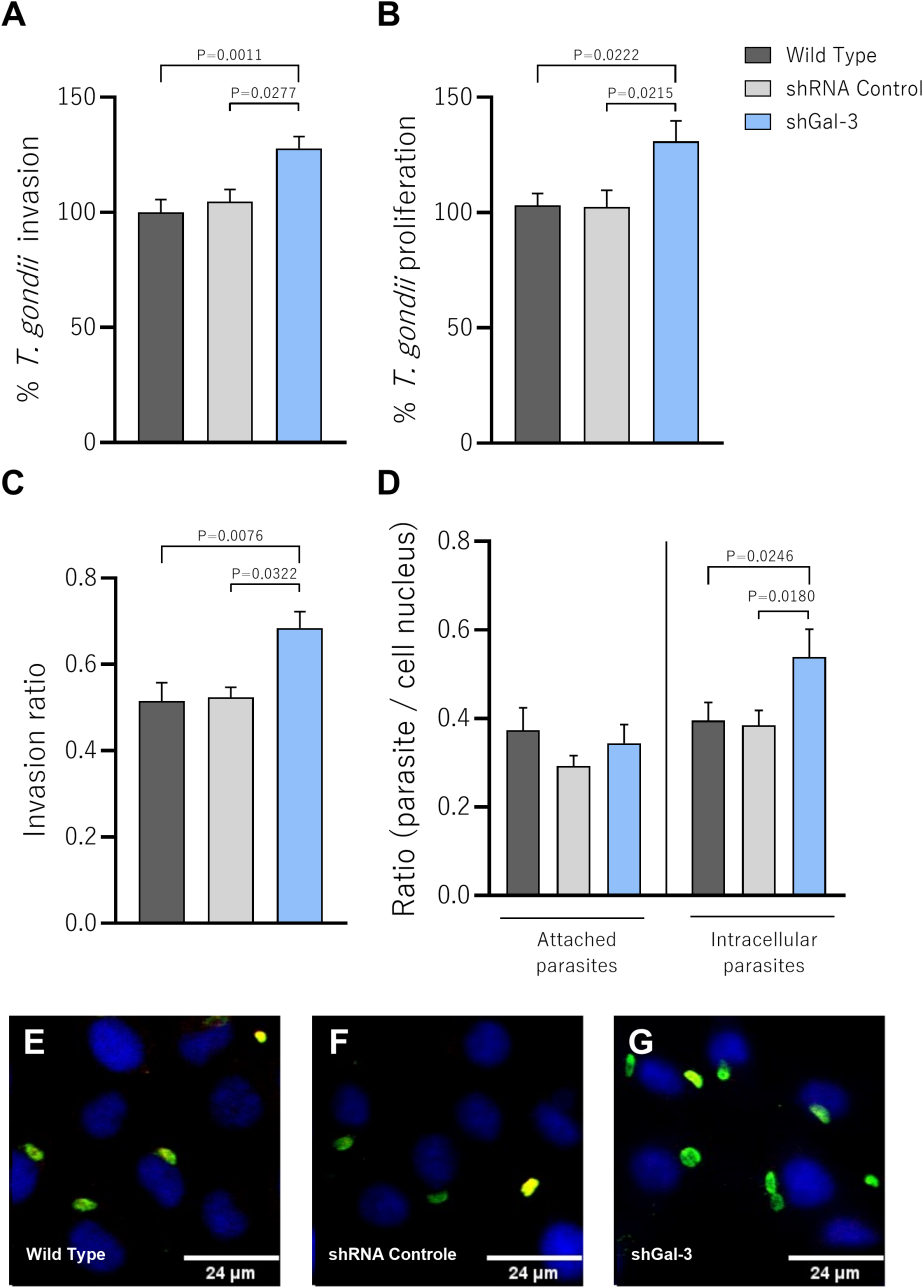
The knockdown of Galectin-3 in BeWo cells increases the invasion and intracellular proliferation of *T. gondii*. **(A)** BeWo cells silenced or not for Gal-3 were infected for 3h to evaluate *T. gondii* invasion or **(B)** for 24h to evaluate parasite proliferation. In both experimental situations, the number of tachyzoites was determined by β-galactosidase assay and expressed as percentage. The proportion of the number of intracellular tachyzoites in relation to the total number of parasites was considered as the invasion ratio **(C)**. The number of intracellular (green^+^/red^−^) and attached [red or red^+^/green^+^ (yellow)] parasites was scored in 20 randomly selected fields and expressed as the ratio of the number of parasites per cell nucleus **(D)**. Representative fluorescence images highlighting the impact of the decrease of Gal-3 expression on parasite proliferation **(E–G)**. Photomicrographs were captured at 40x magnification (Scale bar: 24μm). Data are presented as means ± standard error of the means (SEM). Significant differences were analyzed using one-way ANOVA with Sidak’s multiple comparison post-test. Differences were considered statistically significant when P < 0.05.

After confirming the gene silencing, we checked the impact of the protein knockdown on the invasion and proliferation of *T. gondii* in BeWo cells (Wild Type, shRNA control and shGal-3). We assessed parasite invasion (3h) or intracellular proliferation (24h) using the β-galactosidase assay. We observed that the Gal-3 knockdown increased *T. gondii* invasion in comparison with Wild Type (P=0.0011) or shRNA control (P=0.0277) BeWo cells (**Figure 2A**). Similarly, reduced expression of Gal-3 in BeWo cells favored parasite proliferation compared to Wild Type (P=0.0222) or shRNA control (P=0.0215) BeWo cells (**Figure 2B**), suggesting that this protein appears to be a key protein involved in the parasitism control.

**Figure 3:**
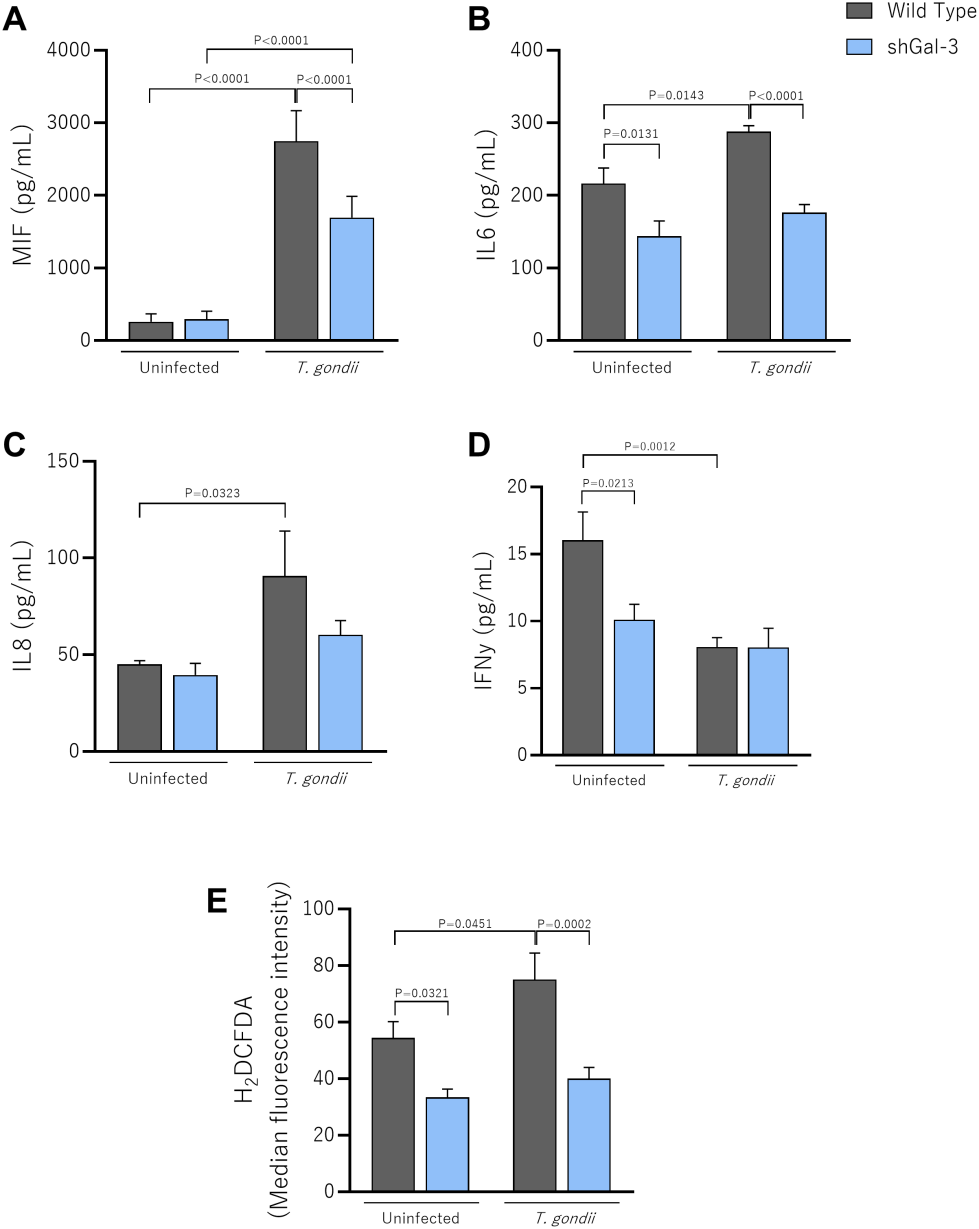
The knockdown of Galectin-3 reduces the production of MIF and IL6 in *T. gondii*-infected BeWo cells. Wild Type or shGal-3 BeWo cells were infected or not with *T. gondii* tachyzoites and cell culture supernatants were collected for measurement of **(A)** MIF, **(B)** IL6, **(C)** IL8 and **(D)** IFNγ. **(E)** Infected cells were incubated with H_2_DCF-DA, and the ROS production was expressed as median fluorescence intensity of H_2_DCFDA. Data are shown as mean ± standard error of the means (SEM). Differences were analyzed using one-way ANOVA with Sidak’s multiple comparison post-test. Differences were considered statistically significant when P < 0.05.

To consolidate our findings and confirm the ability of Gal-3 to interfere in the first steps of parasitic infection, we evaluated the adhesion and invasion rates after 3h of *T. gondii* infection using the green/red assay. Corroborating with our previous findings, knockdown of the target protein in BeWo cells resulted in a significant increase in the invasion ratio compared to Wild Type (P=0.0076) or shRNA control (P=0.0322) BeWo cells (**Figure 2C**). In line with this result, the decreased expression of Gal-3 significantly increased the number of intracellular parasites in relation to the Wild Type (P=0.0246) or shRNA control (P=0.0180) BeWo cells (**Figure 3B**). Curiously, shGal-3 BeWo cells did not show any statistical difference in the number of attached parasites compared to the control groups (**Figure 2D**). Illustrative fluorescence images highlighting attached and intracellular *T. gondii* tachyzoites are demonstrated (**Figures 2E–G**).

### 2.3 Reduced Gal-3 expression downmodulated MIF, IL6 and ROS levels in *T. gondii*-infected BeWo cells

So far, our findings have shown that the reduction of Gal-3 has a direct effect on the parasite proliferation, as they exhibit an increased ability to invade and replicate within host cells. However, we could not exclude the possibility that Gal-3 may affect the host immune response. Therefore, we investigated the potential immunomodulatory effects in the presence or reduction of this protein by measuring cytokines levels. We measured IL4, IL6, IL8, IL10, IFNγ, MIF and TNFα levels in the supernatant of BeWo cells under different experimental conditions.

We observed that *T. gondii* infection promoted a strong upregulation of MIF levels by Wild Type (P<0.0001) and shGal-3 (P<0.0001), compared to the uninfected Wild Type and shGal-3 BeWo cells, respectively. Interestingly, the infected shGal-3 cells had lower MIF production than infected Wild Type cells (P<0.0001) (**Figure 3A**).

Reducing Gal-3 expression induced a decrease in IL6 levels compared to Wild Type cells (P=0.0131). During *T. gondii* infection, there is a strong upregulation of IL6 in Wild Type cells compared to the uninfected condition (P=0.0143), but the levels also remained reduced in shGal-3 cells compared to infected Wild Type cells (P < 0.0001) (**Figure 3B**).

Regarding IL8 production, our data revealed that *T. gondii*-Wild Type had higher IL8 production than uninfected Wild Type (P=0.0323) (**Figure 3C**). On the other hand, IFNγ levels were higher in uninfected Wild Type compared to infected Wild Type (P=0.0012); in addition, in the uninfected condition, shGal-3 cells showed a reduction in secretion of IFNγ compared to Wild Type cells (P=0.0213) (**Figure 3D**). IL4, IL10 and TNFα cytokines were not detected in supernatants from BeWo cells under any experimental conditions (data not shown).

Regarding ROS production, in the absence of infection, our data demonstrated that reduced Gal-3 expression decreased significantly ROS levels compared to BeWo Wild Type (P=0.0321) (**Figure 3E**). As expected, *T. gondii* infection increases ROS production in BeWo Wild Type in comparison to these same uninfected cells (P=0.0451); interestingly, infected BeWo shGal3 cells showed a reduction in ROS levels in relation to BeWo WT cells (P=0.0002) (**Figure 3E**).

Together, these findings suggest that the downmodulation of Gal-3 is associated with reduced levels of essential immune mediators responsible for controlling *T. gondii* infection.

### 2.4 GB1107-mediated inhibition of Gal-3 in human villous explants contributed to the increase in the *T. gondii* proliferation, possibly through the reduction of MIF and IL6 levels

**Figure 4:**
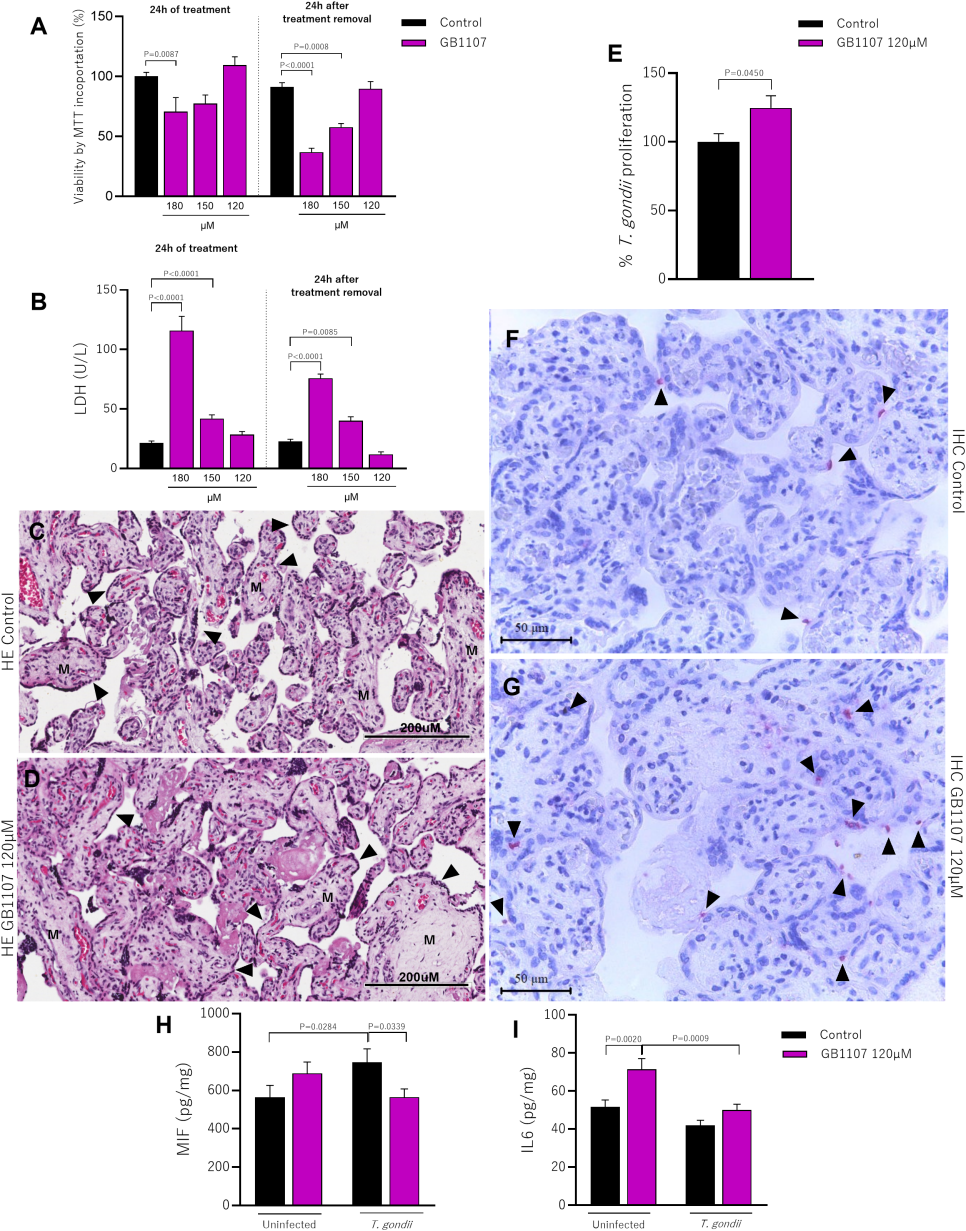
Treatment with synthetic inhibitors (GB1107) does not affect the viability of placental villi and increases parasitism. Placental villi were incubated for 24h with GB1107 (180, 150, 120µM), and subsequently the treatment was removed and only culture medium was added for another 24h and subjected to viability analysis. **(A)** Viability of placental villi was determined by MTT incorporation, with the control group considered as 100% viable. **(B)** Supernatants from placental villi were collected and used to measure LDH levels (U/L). Representative photomicrographs of hematoxylin-eosin (HE) stained histological sections of placental villi treated with **(C)** culture medium or **(D)** inhibitor (120µM) for 24h are shown [syncytiotrophoblast cells (black arrowhead) and mesenchyme (M)]. Photomicrographs were captured at 40x magnification (scale bar: 200µm). **(E)** Placental villi previously incubated with inhibitor (120µM) were infected with *T. gondii* tachyzoites for 24h, and the percentage of intracellular parasite proliferation was measured by the β-galactosidase assay, with untreated/infected (control group) considered as 100% parasite proliferation. **(F, G)** Representative photomicrographs show *T. gondii* tachyzoites (black arrowhead) immunolocated by immunophosphatase staining and counterstained with hematoxylin (scale bar: 50μm). Additionally, placental villi were infected or not with *T. gondii* tachyzoites, and cell culture supernatants were collected for measurement of **(H)** MIF, **(I)** IL6. Data are shown as mean ± standard error of the means (SEM). Differences were analyzed using one-way ANOVA with Sidak’s multiple comparison post-test. Differences were considered statistically significant when P < 0.05.

The viability of placental villi was evaluated after 24h treatment with GB1107, or after its removal for an additional 24h, using the MTT assay. We observed that during the initial 24h of treatment, there was toxicity to the explant only at the concentration of 180µM (P=0.0087), and this toxicity persisted even after treatment removal, showing irreversibility effect (P<0.0001), compared to respective controls (groups incubated only with culture medium); the concentration of 150µM showed toxicity only in the removal condition when compared to the untreated control, suggesting that this is still a high dose and its residue causes damage to the tissue (P=0.0008). Thus, in both experimental conditions analyzed, we observed that only the lowest concentration used (120µM) did not change tissue viability compared to the control group (**Figure 4A**). Confirming our MTT viability assay, the LDH assay also revealed that during the initial 24h of treatment, there was toxicity to the explant at concentrations of 180µM (P<0.0001) and 150µM (P<0.0001), and this toxicity persisted even after treatment removal, showing maintenance of toxicity (180µM P<0.0001; 150µM P=0.0085). As expected, GB1107 was not toxic to placental villi at the concentration of 120µM in both experimental conditions adopted (*i.e.*, 24h treatment or treatment removal) compared to the control group (**Figure 4B**). In addition, morphological analysis for the concentration of 120µM demonstrated the maintenance of tissue integrity highlighted by a typical morphology of syncytiotrophoblast cells (black arrowhead) and mesenchyme (M) compared to the untreated group (**Figures 4C, D**).

Based on these results, we promoted the blockade of Gal-3 in placental villi using a safe and effective concentration of GB1107 (120µM). We evaluated the *T. gondii* intracellular proliferation. Our data demonstrated that the Gal-3 inhibition increased parasitic proliferation compared to untreated placental villi (P=0.0450) (**Figure 4E**). Corroborating with the parasitism assay, the immunohistochemistry showed an increase in the number of *T. gondii* tachyzoites (black arrowhead) after Gal-3 inhibition in comparison with the infected/uninhibited human villous explants (**Figures 4F, G**).

Seeking to understand the impact of Gal-3 inhibition mediated by inhibitor treatment on the immune response, in the presence or absence of infection, we measured cytokine levels in the supernatant of placental villi. We observed that *T. gondii* infection increased MIF levels in untreated villi (P=0.0284) compared to the uninfected control. Interestingly, infected villi receiving the inhibitor had lower MIF production than untreated infected villi (P=0.0339) (**Figure 4H**). The reduction in Gal-3 expression led to an increase in IL6 levels compared to untreated villi (P=0.0020); however, during *T. gondii* infection, IL6 is downregulated in inhibitor-treated villi compared to uninfected condition (P=0.0009) (**Figure 4I**).

## 3 Materials and methods

### 3.1 Cell culture and parasite maintenance

Human villous trophoblast cells (BeWo lineage) were commercially purchased from the American Type Culture Collection (ATCC, Manassas, VA, USA) and maintained in RPMI 1640 medium (Cultilab, Campinas, SP, Brazil) supplemented with 100U/mL penicillin (Sigma Chemical Co., St. Louis, MO, USA), 100μg/mL streptomycin (Sigma) and 5% heat-inactivated fetal bovine serum (FBS) (Cultilab). Cultures were kept at 37°C under a humidified atmosphere containing CO_2_ (5%). *Toxoplasma gondii* tachyzoites (highly virulent RH strain, 2F1 clone) constitutively expressing the β-galactosidase gene were maintained by serial passages in BeWo cells cultured in RPMI 1640 medium containing 2% FBS, 100U/mL penicillin and 100μg/mL streptomycin at 37°C and CO_2_ (5%) (Teixeira et al., 2023a).

### 3.2 Galectin-3 shRNA transfection

We promoted the Gal-3 knockdown in human villous trophoblast cell lines (BeWo lineage) using human shRNA lentiviral particles, according to the manufacturer. Briefly, 0.5x10^5^ cells were plated in 6-well culture plates in a final volume of 1.5mL and incubated at 37°C and 5% CO_2_ for 18h. Next, cells reached 50% confluency and were transfected with 10μL of Galectin-3 shRNA (h) lentiviral particles (SC-155994-V, Santa Cruz Biotechnology, INC) or control shRNA lentiviral particles (SC-108080) in 10% FBS medium containing Polybrene (5µg/mL) (SC-134220) and incubated for 18h at 5% CO_2_ and 37°C. Following, the medium was changed to medium containing only 10% FBS and the cells were allowed to proliferate for additional 24h. Afterwards, BeWo cells were treated with 3µg/mL of puromycin (SC-108071) to select clones expressing shRNA. Medium containing puromycin (3µg/mL) was replaced every 48h for around 10 days. Finally, selected clones were expanded and tested to determine Gal-3 expression using the western blotting and immunofluorescence techniques.

### 3.3 Western blotting

To confirm whether the Gal-3 expression was partially silenced, we performed the western blotting assay, as previously described (Barbosa et al., 2015). Briefly, the PVDF membrane was blockaded, and incubated for 18h with the rat monoclonal antibody anti-Gal-3 (1:1000, 14-5301-82, BioM3/38, eBioscience), followed incubation with the HRP-conjugated anti-rat IgG secondary antibody (1:2000, Jackson ImmunoResearch Laboratories, West Grove, PA, USA). The expression of β-actin was used as a control (1:1000; SC-81178; Santa Cruz Biotechnology, INC). Finally, the membrane was visualized using the chemiluminescence kit (Thermo Scientific) on the ChemiDoc MP Imaging System (BIO-RAD Laboratories, Inc., Hercules, CA, USA) for Gal-3 or β-actin detection.

### 3.4 Immunofluorescence assay

Gal-3 expression was also verified by immunofluorescence. BeWo cells (1x10^5^/500µl/well) were plated on 24-well microplates containing 13-mm coverslips and incubated at 37°C and CO_2_ (5%) for 18h. Subsequently, cells were fixed with 4% paraformaldehyde (PFA) for 45min at room temperature, then washed with 1x PBS and incubated for 18h with the rat monoclonal antibody anti-Gal-3 [diluted 1:1000 in PGN (PBS containing 0.25% gelatin + 0.01% saponin)] (14-5301-82, BioM3/38, eBioscience)] in the dark at 4°C. After the incubation period, coverslips were washed with 1x PBS and incubated with anti-rat IgG conjugated to Alexa Fluor 488 (green fluorochrome) and TO-PRO-3 (blue fluorochrome) (Invitrogen California, USA) (both diluted 1:500 in PGN 0.01% saponin) for 1h in the dark at 4°C to label Gal-3 and the nucleus, respectively. Coverslips were mounted onto glass slides and samples were analyzed using confocal fluorescence microscopy (40X, Zeiss, LSM 510 Meta, Germany) with an inverted microscope (Zeiss Axiovert 200 M). The mean of Galectin-3 fluorescence was determined by setting a high threshold in ImageJ software (National Institutes of Health, USA).

### 3.5 *T. gondii* invasion and intracellular proliferation by β-galactosidase activity

Wild Type, shRNA control and shGal-3 BeWo cells (3x10^4^/100µL/well) were cultured in 96-well microplates containing RPMI medium and 10% FBS, and kept for 18h in an incubator at 37°C with 5% CO_2_. Subsequently, the cells were infected with *T. gondii* tachyzoites at a multiplicity of infection (MOI) of 3:1 (ratio of parasites per cell), in RPMI medium with 10% FBS for 3h to assess *T. gondii* invasion or for 24h to evaluate *T. gondii* intracellular proliferation. Furthermore, culture supernatants from the proliferation assay were collected and stored at −80°C for further cytokine determination. The parasite invasion and intracellular proliferation were assessed using the colorimetric β-galactosidase assay, a reaction using the chlorophenol red-β-D-galactopyranoside reagent substrate (CPRG; Roche Diagnostics, Mannheim, Germany), as previously described (Teixeira et al., 2020).

### 3.6 Invasion and attachment assay

Wild Type, shRNA control and shGal-3 BeWo cells (1x10^5^/500μL/well) were seeded in 24-well microplates containing 13-mm coverslips. Afterwards, the cells were infected with *T. gondii* tachyzoites (3:1) for 3h, rinsed twice with 1x PBS, and then fixed with 4% PFA for 15min at room temperature. The cells were incubated with rabbit polyclonal primary anti-*T. gondii* antibody (Abcam #20530; Waltham, MA, USA) [diluted 1:500 in PGN (PBS containing 0.25% gelatin)] at room temperature for 1h, followed by Alexa Fluor 594-conjugated anti-rabbit IgG (Invitrogen, USA #A11012; Waltham, MA, USA) also diluted 1:500 in PGN. Next, cells were incubated for 1h with rabbit polyclonal primary anti-*T. gondii* antibody (diluted 1:500 in PGN-0.01% saponin - permeabilizing solution) followed by incubation for 1h with Alexa Fluor 488-conjugated anti-rabbit IgG (Invitrogen, USA #A11008; Waltham, MA, USA) and the cell nucleus marker DAPI (Invitrogen, USA), both diluted 1:500 in PGN + saponin. Coverslips were mounted onto glass slides and samples were analyzed using confocal fluorescence microscopy (40X, Zeiss, LSM 510 Meta, Germany) with an inverted microscope (Zeiss Axiovert 200 M). The number of intracellular (green^+^/red^−^) and adhered [red or red^+^/green^+^ (yellow)] parasites were scored on 20 randomly-selected fields on each separately-mounted coverslip (de Melo Fernandes et al., 2023; Teixeira et al., 2023a). The proportion of the number of intracellular tachyzoites to the total number of parasites was considered as the invasion ratio. Two independent experiments with four replicates were performed.

### 3.7 Human chorionic villous explant culture

To corroborate with our *in vitro* results, we used third-trimester human placental villous explants as an *ex vivo* model, which is a widely used experimental model of the maternal-fetal interface (Costa et al., 2020; Rosini et al., 2022; Martínez et al., 2023; Teixeira et al., 2023b). Firstly, third-trimester human placentas (36 to 40 weeks of pregnancy, N = 5) were collected after elective cesarean section deliveries at the Clinics Hospital of the Universidade Federal de Uberlândia (HC-UFU), MG, Brazil. The experimental methods were approved by the pertinent Ethics Committee with approval number 5.614.590.

Placental tissues were collected following exclusion criteria as previously described (Teixeira et al., 2020). Briefly, terminal chorionic villi containing five to seven free tips per explant were collected as described previously and added to 96-well microplates (one villus per well) in 200µL/well of a fresh RPMI 1640 medium 10% FBS for 24h at 37°C under a humidified atmosphere containing CO_2_ (5%) until the experimental procedure.

### 3.8 Viability of human chorionic villous explant

To impair the Gal-3 activity in the villi tissue, we used a specific inhibitor to Gal-3 (Zetterberg et al., 2018; Vuong et al., 2019), which penetrates cells and blocks protein expression, GB1107 (3,4-dichlorophenyl 3-deoxy-3-[4(3,4,5-trifluorophenyl)-1H-1,2,3-triazol-1-yl]-1-thio-α-D-galactopyranoside) - kindly provided by Galecto Biotech (Boston, Massachusetts, USA). According to the literature, the villi explants were subjected to two distinct treatment protocols with GB1107 (at concentrations of 120, 150 and 180µM): (I) continuous treatment for 24h, and (II) a 24-hour treatment period with GB1107, followed by the treatment removal and replacement with treatment-free medium. Next, tissue viability was measured by MTT, LDH and histologic assays, according to published protocols (Teixeira et al., 2020). Three independent experiments with eight replicates were performed. Furthermore, we performed morphological analysis of treated villi to corroborate the viability assays. Villi tissue sections were stained with hematoxylin/eosin and examined using a light microscope (X40, Scanscope AT). One independent experiment with three replicates was performed.

### 3.9 *T. gondii* infection of human chorionic villous explants

We quantified *T. gondii* intracellular proliferation in human villous explants treated or not using a colorimetric β-galactosidase assay. Briefly, villi were collected and cultured in 96-well microplates (one villus/200µL/well) in a supplemented culture medium for 24h at 37°C and 5% CO_2_. Next, the villi were treated with GB1107 (120µM) for 24h and then infected with *T. gondii* tachyzoites of RH strain-2F1 (1×10^6^ parasites/200µL/well) and incubated for 24h at 37°C and 5% CO_2_ villous explants were collected and stored at − 80°C for the following analyses: protein content determination using Bradford reagent and *T. gondii* intracellular proliferation by β-galactosidase assay according to published protocols (Teixeira et al., 2020). Complementing the β-galactosidase assay, treated and infected villous explants were submitted to an immunohistochemistry assay to verify the immunolocalization of the parasites as previously described (de Melo Fernandes et al., 2023). Three independent experiments with eight replicates were performed.

### 3.10 Cytokine measurement

The levels of the human cytokines IL4, IL6, IL8, IL10, TNFα, IFNy and MIF released in culture supernatants, produced by BeWo cells or human villous explants under the different experimental conditions, were measured using a double-antibody sandwich enzyme-linked immunosorbent assay (ELISA), following the manufacturer’s instructions (BD Bioscience, San Diego, CA, USA; R&D Systems, Minneapolis, MN, USA). The data were expressed in pg/mL according to a standard curve for each cytokine for BeWo cells. For placental explants, cytokine concentrations were normalized using a ratio between cytokine production (pg/mL) and its corresponding total protein content (μg/mL) of each sample, with levels shown in pg/mg of tissue.

### 3.11 Intracellular reactive oxygen species measurement

The determination of ROS levels produced by BeWo cells under different experimental conditions was based on the intracellular peroxide-dependent oxidation of 2′,7′-dichlorodihydrofluorescein diacetate (H_2_DCF-DA) (Invitrogen, #D399) to form the fluorescent compound 2′,7′-dichlorofluorescein (DCF), as previously described (de Melo Fernandes et al., 2023), with some modifications. In brief, Wild Type and shGal-3 BeWo cells (3x10^4^/100μL/well) were seeded in black 96-well microplates with clear bottoms (Costar REF# 3603, New York, USA). After adhesion, cells were infected or not with *T. gondii* tachyzoites (MOI of 3:1) for 24h at 37°C and 5% CO_2_. As control of the reaction, cells were incubated with 3.5% hydrogen peroxide (H_2_O_2_) diluted in 1× PBS. Afterward, cells were incubated with 150μL of H_2_DCF-DA (10μM; diluted in 1× PBS containing 10% FBS) for 45min under darkness at 37°C and 5% CO_2_. Lastly, cells were rinsed with 1× PBS and the microplate submitted to an excitation (488nm) and emission (550nm) wavelength in the multi-well scanning spectrophotometer (Versa Max ELISA Microplate Reader, Molecular Devices, Sunnyvale, CA, EUA). The data were presented as mean fluorescence intensity (MFI).

## 4 Discussion

Gal-3 is a multifunctional β-galactoside-binding lectin associated with implantation, embryogenesis, placental formation, as well as maintenance and success of pregnancy (Yang et al., 2011, 2013). Interestingly, it has been reported that Gal-3 plays a prominent participation during infections by protozoan parasites, including *T. gondii* (Alves et al., 2010, 2013). In this scenario, we are the first to investigate the role of Gal-3 during *T. gondii* infection through well-established and widely experimental models of maternal-fetal interface (Costa et al., 2020; Rosini et al., 2022; Martínez et al., 2023; Teixeira et al., 2023b).

To unravel whether Gal-3 can modulate essential steps of the *T. gondii* infection, we assessed the adhesion, invasion and proliferation processes. Our data revealed that reduced expression of Gal-3 in BeWo cells and human villous explants favored parasite infection, which was highlighted by higher ratios of invasion and intracellular proliferation, indicating that this lectin plays an important role in the parasitism control. Corroborating with our data, Bernardes et al. (2006) demonstrated that infection with the ME-49 strain of *T. gondii* in Gal-3^-/-^ mice exhibited a higher parasite burden in the brain (Bernardes et al., 2006). In infections by other protozoa such as *Trypanosoma cruzi*, the lack of Gal-3 increased replication *in vitro* and systemic parasitemia *in vivo* (Chain et al., 2020). Gal-3 also plays a role in controlling invasion, replication and endocytic vesicle formation in *Leishmania amazonensis* infection (Oliveira et al., 2021).

Our findings demonstrated that reduced Gal-3 expression increased the susceptibility to *T. gondii* infection. Hence, the following question was raised: would the deficiency of Gal-3 expression be able to modulate the expression of immunological mediators important in the parasitism control? The literature has already demonstrated the importance of Gal-3 in the immunological response against *T. gondii* and other parasites. It has shown that Gal-3 expressed in macrophages recognizes glycosylphosphatidylinositols (GPIs) on the surface of *T. gondii*, an interaction necessary to generate an effective anti-parasitic immune response (Debierre-Grockiego et al., 2010). The binding of Gal-3 to the GPIs of *T. gondii* increases TNFα levels in infected macrophages and, in parallel, Gal-3 can also act as a co-receptor, presenting the GPIs to toll-like receptors (TLRs) on macrophages (Debierre-Grockiego et al., 2010).

Gal-3 can develop a different immune response according to the experimental model adopted (Liu et al., 2018). After infection with the ME-49 strain of *T. gondii*, Gal-3^-/-^ mice exhibited a higher parasite burden, decreased recruitment of monocytes/macrophages and neutrophils, delayed inflammatory response in the central nervous system and significantly showed higher concentrations of IL12 and IFNγ in serum compared to Gal-3^+/+^ mice, suggesting that Gal-3 is an important molecule in the course of an immune response to control *T. gondii* proliferation *in vivo* (Bernardes et al., 2006).

Regarding other protozoan parasites, Gal-3 is responsible for recognizing lipophosphoglycan present on the surface of *L. major*, suggesting that the molecule may contribute to a specific immune response against leishmaniasis (Pelletier and Sato, 2002). Another study demonstrated that Gal-3 recognizes glycans present in *S. mansoni*, which may be a pattern for the parasite’s immunological recognition mediated by this galectin (van den Berg et al., 2004). In addition, this molecule appears to be important in the control of *T. cruzi* which, by evading immunological mechanisms triggered by Gal-3 during infection, alters the molecular structure of galectin, thus nullifying the signaling mechanisms associated with innate immunity that are triggered by this lectin (Pineda et al., 2015, 2020).

Thus, we evaluated the immunomodulatory effects of Gal-3 by measuring cytokine levels present in the supernatants from Wild Type and shGal-3 BeWo cells or in human villous explants infected or not by *T. gondii*. In general, we observed that the downmodulation of Gal-3 reduced the MIF and IL6 production in *T. gondii*-infected experimental conditions. In the context of the maternal-fetal interface, previous studies from our research group have extensively reported the role of IL6 and MIF during *T. gondii* infection (Rosini et al., 2022; de Melo Fernandes et al., 2023; Teixeira et al., 2023a). It has been demonstrated that higher MIF levels in BeWo cells and villous explants infected by *T. gondii* are associated with parasite clearance (de Oliveira Gomes et al., 2011; Franco et al., 2011). In a similar way, IL6 plays an important role during embryo invasion and implantation in the uterus (Champion et al., 2012) and is involved in the control of infections by intracellular parasites, especially *T. gondii* (Barbosa et al., 2015; Gomes et al., 2018; de Melo Fernandes et al., 2023; Teixeira et al., 2023a). Taken together, we demonstrated that the low IL6 and MIF levels in shGal-3 BeWo cells could be responsible for the impairment of the *T. gondii* invasion and proliferation, as previously described in distinct models at the maternal-fetal interface (de Melo Fernandes et al., 2023; Teixeira et al., 2023a).

Our findings demonstrate that Gal-3 plays a key role in regulating ROS production in BeWo cells. Reduced Gal-3 expression in BeWo cells significantly impairs ROS levels even under infection. These findings are corroborated by Alves et al. (2013), which highlights the importance of Gal-3 in ROS generation during acute *T. gondii* infection, crucial for controlling parasite growth. Therefore, our data reinforce the role of Gal-3 in ROS production as a possible defense mechanism against parasite infection.

Our findings indicate that the downmodulation of Gal-3 is associated with a reduced level of cytokine production, and that this lectin would be fundamental in contributing to the secretion of important cytokines in the cellular immune response against the pathogen at the maternal-fetal interface. Our study sheds light on the role of Gal-3 in congenital toxoplasmosis; however, it is worth mentioning that previous studies have addressed that Gal-3 is also relevant in chronic *T. gondii* infection. Bernardes et al. (2006) observed that, 14 days after infection, the number of cysts in the brains of Gal-3^-/-^ mice was 11 times higher than in Gal-3^+/+^ mice. These data strongly suggest that the absence of Gal-3 facilitates parasite replication and persistence, potentially by disrupting key immune processes that typically control cyst formation in the central nervous system. Therefore, Gal-3 could represent a therapeutic target or biomarker for assessing susceptibility to *T. gondii* infection; by modulating Gal-3 pathways, it might be possible to control parasite persistence, offering new avenues for treatment.

In conclusion, our results highlight that Gal-3 is an important protein for controlling the invasion and intracellular proliferation of *T. gondii* in BeWo cells and human villous explants, since its deficiency is related to a reduction of MIF, IL6 and ROS, pro-inflammatory immune mediators important for controlling parasitic infection. Studies are still needed to investigate other mechanisms that this lectin uses to play this role in the placental microenvironment so that we can understand its function in controlling the vertical transmission of the parasite.

## Data Availability

The original contributions presented in the study are included in the article/supplementary material. Further inquiries can be directed to the corresponding author.
